# Intraoperative Imaging with a Portable Gamma Camera May Reduce the False-Negative Rate for Melanoma Sentinel Lymph Node Surgery

**DOI:** 10.1245/s10434-018-6685-1

**Published:** 2018-08-13

**Authors:** Stanley P. Leong, Max Wu, Ying Lu, Donald M. Torre, Anna von Bakonyi, Arianna M. Ospina, James D. Newsom, William S. Luckett, Christopher W. Soon, Kevin B. Kim, Mohammed Kashani-Sabet

**Affiliations:** 1 California Pacific Medical Center, San Francisco, CA USA; 2 Stanford University, Stanford, CA USA

## Abstract

**Background:**

Preoperative imaging and intraoperative gamma probe (GP) localization is standard for identifying sentinel lymph nodes (SLNs) in melanoma patients. The aim of this prospective Institutional Review Board-approved study was to investigate whether an intraoperative portable gamma camera (PGC) improves SLN detection over the GP.

**Methods:**

Lymphoscintigraphy and single photon emission computed tomography/computed tomography were performed after injection of 99mTc-Tilmanocept in melanoma patients (≥ 18 years, Breslow thickness ≥ 1.0 mm). A GP was used to localize the SLNs in each basin, which was explored by the GP to ensure that the operative field was < 10% counts of the hottest SLN. The PGC was then used after a negative GP screening. Any residual hotspots identified by the PGC were considered as additional SLNs and were removed following the 10% rule.

**Results:**

Preoperative imaging of 100 patients identified 138 SLN basins, with 306 SLNs being identified by conventional surgery. The PGC localized 89 additional SLNs in 54 patients. Thus, the PGC identified an additional 23% of SLNs [95% confidence interval (CI) 18–27%]. Four of these 89 SLNs showed micrometastasis in four patients, in two of whom the only tumor-positive SLN was identified by the PGC, preventing two false-negative cases. Thus, the null hypothesis that the PGC did not detect additional positive SLNs was rejected (*p* = 0.000). The overall SLN positive rate was 9.9% (39/395, 95% CI 6–12), and the overall patient positive rate was increased using the PGC, from 25 to 27% (27/100).

**Conclusions:**

Intraoperative PGC imaging yielded additional SLNs in a significant number of patients over GP alone. Identification of these additional SLNs resulted in upstaging of four patients with two patients being converted from a negative to a positive status, thus, preventing two false-negative cases.

The incidence of melanoma is rising worldwide.^1^ Selective sentinel lymph node dissection (SSLND) has been recommended for staging patients with primary melanoma with T1b lesions or beyond^2^ according to the 8th edition of the American Joint Committee on Cancer (AJCC).^3^ The MSLT-I study showed that the status of the SLN was the strongest predictor of melanoma-specific survial.^4^ Preoperative lymphoscintigraphy after an intradermal radiotracer injection is required for sentinel lymph node (SLN) mapping. Although an intraoperative handheld gamma probe (GP) is used widely in detecting SLNs, there are several limitations: (1) its usefulness is highly operator-dependent; (2) SLNs can be missed due to an unexpected topographic position; (3) SLNs close to the injection site can be also missed due to the ‘shine-through’ effect; and (4) SLNs may be too small and escape detection by a GP due to its limited field of detection. These limitations may increase the false-negative rate (FNR). Retrospective studies using a high-resolution intraoperative portable gamma camera (PGC) have proven to be of added value for SLN localization.^5^^–^7 A PGC provides real-time imaging with a larger field of view than a GP, image documentation, and visual assistance. Further, it confirms SLN removal. Larger prospective studies are necessary to evaluate the clinical value of PGCs in melanoma SSLND.^8^ Consequently, the aim of this prospective study was to investigate whether a PGC improves the intraoperative SLN identification rate versus GP use alone.

## Materials and Methods

### Patients

This prospective, open-label, single-arm clinical trial (ClinicalTrials.gov identifier: NCT02416336) approved by the California Pacific Medical Center Institutional Review Board was conducted between July 2015 and March 2017. After written informed consent was obtained, 100 patients meeting the following criteria were included: (1) age ≥ 18 years; (2) melanoma (Breslow thickness ≥ 1.0 mm); and (3) no clinically palpable lymph nodes.

### Preoperative Procedure

Lymphoscintigraphy was performed after intradermal injection of 600 µCi (same-day protocol) or 2000 µCi (next-day protocol) of 99mTc-Tilmanocept (Lymphoseek; Cardinal Health, Dublin, OH, USA) in 0.5 mL, divided into four equal aliquots around the primary melanoma or biopsy site. A dynamic study (20 frames at 30 s/frame) was performed immediately following the injection of the radiotracer. Planar imaging was performed at 15 min post-injection. Hybrid single photon emission computed tomography (SPECT) and low-dose computed tomography (CT) images were made 45 min post-injection to localize the SLNs. SPECT acquisition (matrix 64 × 64, 30 frames at 30 s/view) was performed with low-dose CT (140 kV, 2.5 mA, 256 matrix). The fused SPECT/CT images were viewed in the axial, sagittal, and coronal orientation. Planar imaging was repeated after the SPECT/CT acquisition, with the patient being placed in the surgical position. The SLNs were localized and the skin was marked. Involved lymph node basins and number of SLNs were recorded.

### Intraoperative Procedure

**Fig. 1:**
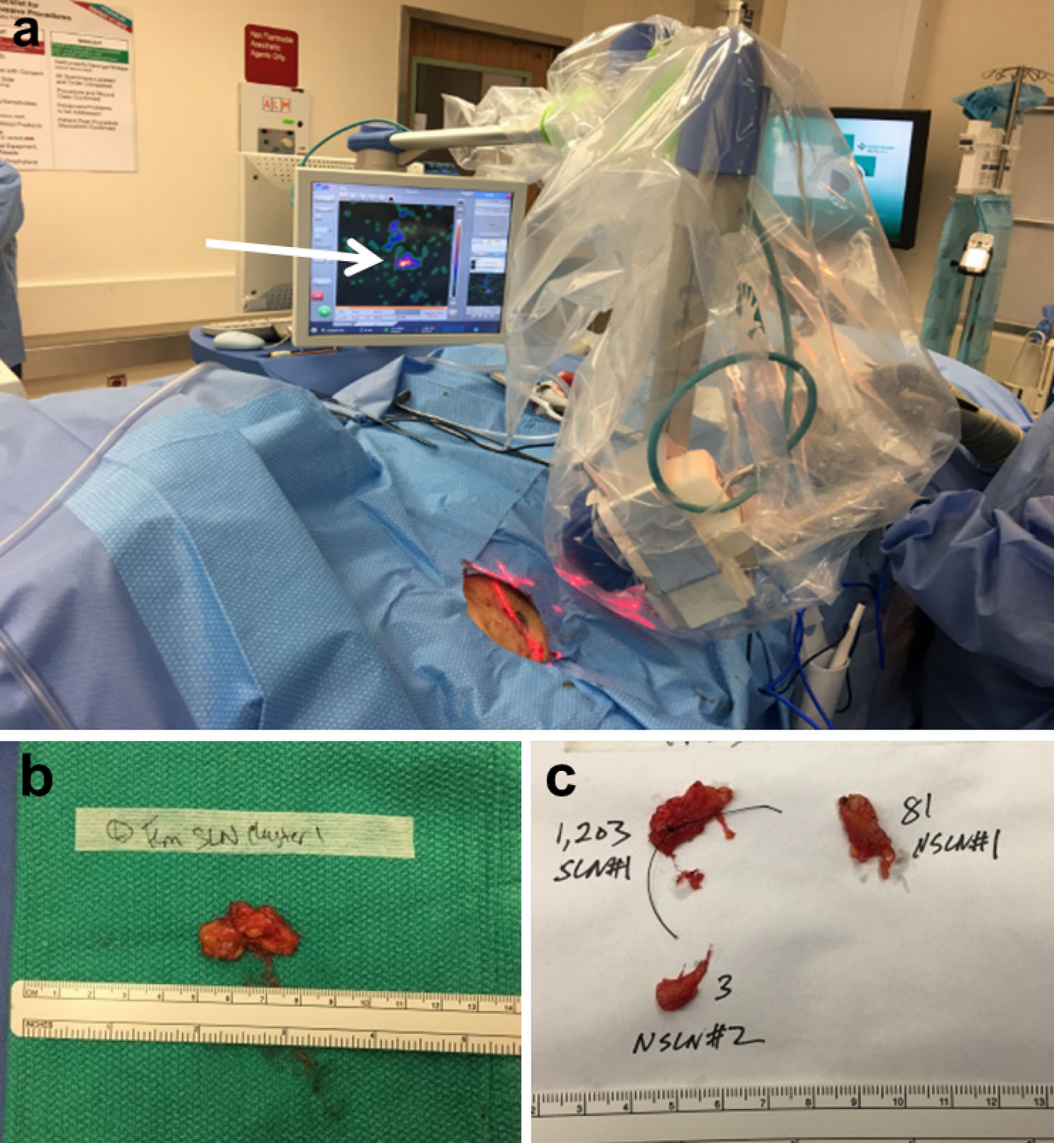
The PGC was draped sterilely and placed above the surgical field, after conventional surgery was completed. The PGC identified a residual lymph node as a hotspot (**a**) on the screen, see arrow. Whether the hot SLN cluster was localized by the GP or found by PGC, the SLN cluster (**b**) was dissected ex vivo, yielding one SLN with a high GP count of 1203 and two non-SLNs with lower counts, which are less than 10% of the hottest SLN (**c**). A black stich was placed in the SLN, where the highest count was detected to allow the pathologist to target this area for microscopic evaluation (**c**). *PGC* portable gamma camera, *GP* gamma probe, *SLN* sentinel lymph node

SSLND was performed by one surgeon (SPL). After excision of the primary melanoma, each identified draining nodal basin was explored using the following steps: (1) a handheld GP (Neoprobe 2000; Neoprobe Corporation, Dublin, OH, USA) was used to guide the incision; (2) the SLN was removed, with in vivo and ex vivo GP counts being recorded; (3) any lymph node in the resection bed with an in vivo count > 10% of the hottest removed SLN or digitally palpable was then excised 9^,^^10^ (when the ex vivo count of this second lymph node was higher than the first node, it was considered as the ‘hottest’ lymph node); (4) a ‘roaming count’ at eight positions of the clock and the center was performed using the GP for a negative screening (equal or similar to radioactive background count); and (5) the PGC (Sentinella S102, Oncovision S.A., Valencia, Spain) was used after ambiguous (approximately 10% of the hottest lymph node) or negative (< 10% of the hottest lymph node) GP screening plus a negative digital palpation (Fig. 1). Steps 1 through 4 constitute conventional surgery. Intraoperatively, the PGC was operated by a Nuclear Medicine Technician. The procedure was terminated following a negative GP and PGC screening. This FDA-approved camera is equipped with a 4 mm pinhole collimator and a CsI(Na) continuous scintillating crystal. The PGC was positioned 5–7 cm, corresponding to a field of view of 6.7 × 6.7 cm to 9.3 × 9.3 cm,^11^ above the resection bed, with an image acquisition time of at least 30 s. Any radioactive hotspots localized in vivo by a laser pointer were considered as additional SLNs detected only by the PGC. The tissue was further explored using the GP to locate and remove additional SLNs that were initially unidentified by conventional surgery. PGC imaging was scored negative when no radioactive hotspots were depicted. All intraoperative data were recorded.

### Ex Vivo Dissection

Each ‘hot’ SLN and its adjacent tissue was termed an SLN cluster, removed and set aside on a separate sterile table. Ex vivo dissection was performed to dissect all lymph nodes contained in each cluster. Lymph nodes with an ex vivo count < 10% were considered non-SLNs, which were only removed as they were part of an SLN cluster specimen. Each SLN and non-SLN was separated according to its ex vivo radioactive count (Fig. 1) and submitted separately to the Pathology Department in a 10% formalin container with its corresponding radioactive count.

### Pathology

All harvested SLNs were fixed in formalin, serially sectioned at 2 mm intervals, and submitted in cassettes for paraffin embedding. Unstained slides were cut at 40 µm intervals for preparation of two flanking hematoxylin and eosin (H&E) slides and four slides for immunohistochemical stains (Melan A, HMB45, S100, and negative control; Ventana Medical Systems, Tucson, AZ, USA; Benchmark Ultra Stainer) for histologic examination. All harvested non-SLNs were sectioned once for H&E staining only.

### Statistical Design and Analysis

An incremental detection rate (IDR) is defined as the proportion of patients who have tumor-positive SLNs detected by PGC, but missed by conventional method. The statistical null hypothesis of this study is an IDR of zero, while the alternative hypothesis is an IDR of 2% or more. The null hypothesis will be rejected if any additional patients with tumor-positive SLNs are observed. The type I error rate is zero under this test. A sample size of 100 patients was chosen for an 85% power to reject null hypothesis when IDR was 2%. Statistical analysis was performed using R version 3.0.3 (The R Project for Statistical Computing, Vienna, Austria). Ninety-five percent statistical confidence intervals (95% CI) were derived for proportions per patient based on normal approximation of binomial distribution. A quasi-Poisson log-linear regression (R-glm) was used to evaluate the relationship between SLN positivity and clinical/histological characteristics.^12^

## Results

### Preoperative Results

**Fig. 2:**
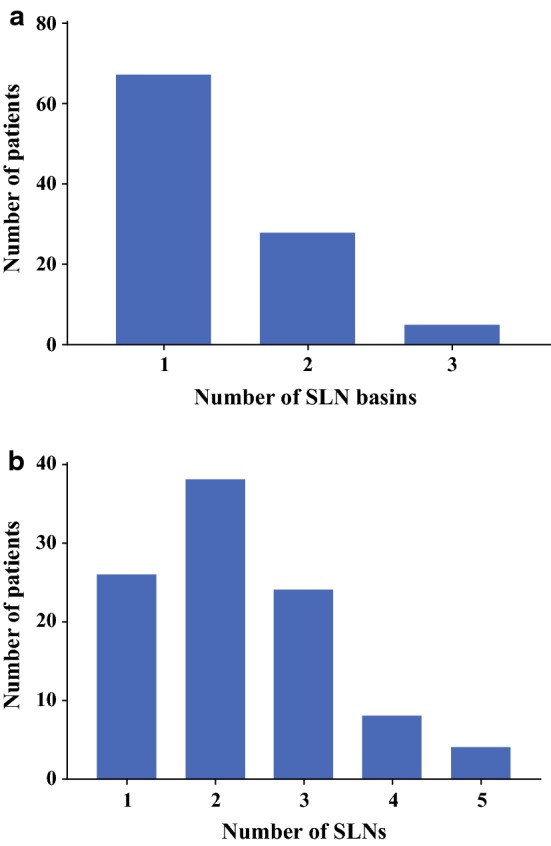
Preoperative visualization of SLN basins (**a**) and number of SLNs (**b**) in 100 patients of this study. *SLN* sentinel lymph node

The 1-day protocol was performed in 58 patients, and the 2-day protocol was performed in 42 patients. A total of 226 SLNs in 138 nodal basins were preoperatively identified in 100 patients (average of 2.3 preoperative SLNs per patient; range 1–5 SLNs) (Fig. 2). In all patients, a SPECT/CT scan was acquired, providing useful anatomic landmarks for planning the surgical procedure. In 13 patients, aberrant drainage was seen (11 in-transit SLNs, 2 epitrochlear SLNs, 1 popliteal SLN).

### Intraoperative Results

**Fig. 3:**
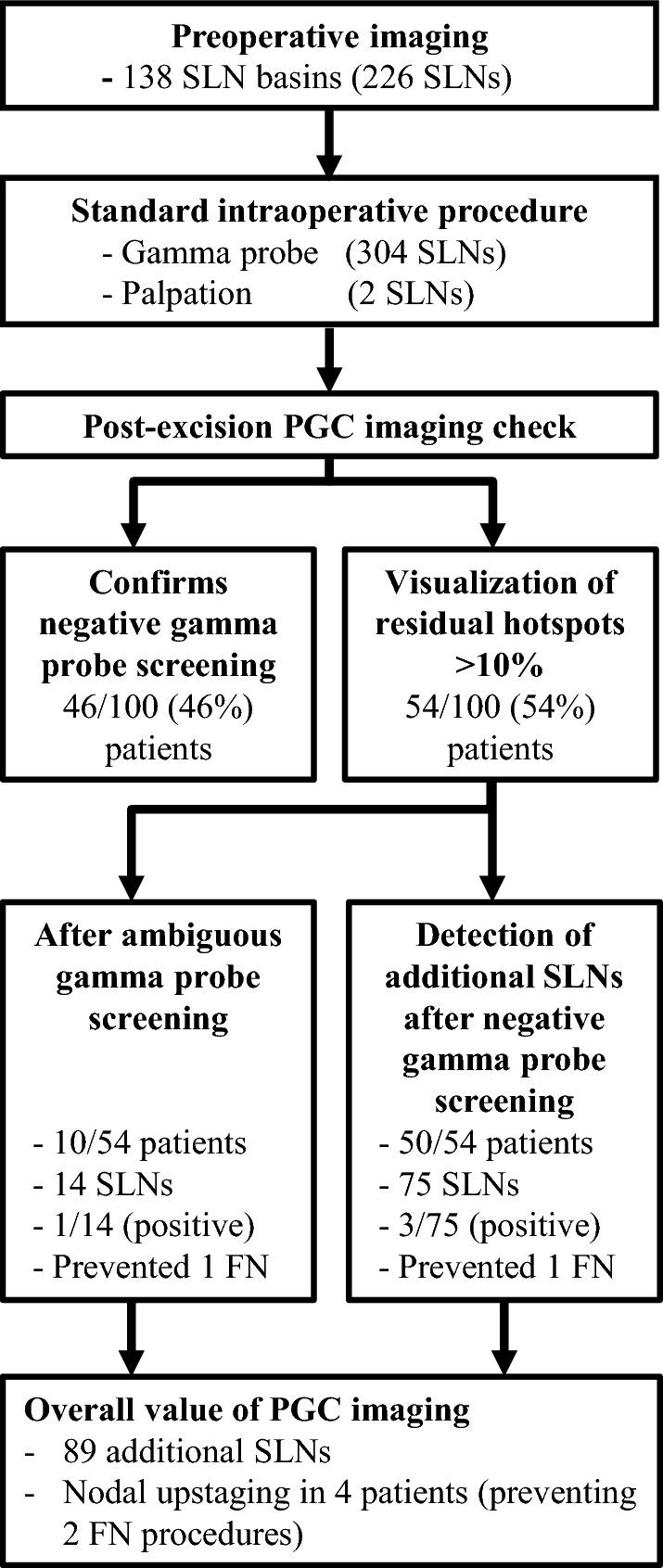
Flowchart visualizing the overall added value of the PGC. The PGC detected 89 SLNs, after ambiguous or negative gamma probe screening plus a negative digital palpation, in 54% of all patients. The removal of these 89 additional SLNs prevented two FN cases, and upstaged four patients from one to two positive lymph nodes in two patients, and from zero to one positive lymph node in the remaining two patients. *PGC* portable gamma camera, *SLN* sentinel lymph node, *FN* false-negative

Patient characteristics are presented in Table 1. All 226 preoperatively identified SLNs could be intraoperatively localized using the GP. Conventional surgery using the GP and palpation resulted in the removal of 306 SLNs, which yielded an average of 3.06 SLNs per patient (95% CI 2.66–3.50) (Fig. 3). The PGC localized 89 additional SLNs in 54 patients, with an increased SLN identification rate of 0.89 per patient (95% CI 0.69–1.13), a significant increase from zero (*p* < 0.0001). In 46/100 patients, PGC imaging confirmed a negative GP screening, while in 10/100 patients, PGC imaging was helpful in identifying 14 SLNs after an ambiguous GP reading. In 50/100 patients, PGC imaging detected 75 additional SLNs after a negative GP screening. The total number of SLNs identified intraoperatively was 395 (306 + 89). The standard procedure alone, including preoperative lymphoscintigraphy, SPECT/CT imaging, intraoperative GP use, plus digital palpation, identified 77% (306/395, 95% CI 73–82) of all SLNs identified. Thus, PGC has identified an additional 23% (95% CI 18–27) of SLNs, which would be missed using conventional procedures.

**Table 1:** Patient characteristics

	Frequency
Sex	
Male	60
Female	40
Age, years [mean (range)]	62 (29–93)
Primary melanoma site	
Trunk	38
Upper extremity	35
Lower extremity	25
Head/neck	1
Dermal metastasis^a^	1
Breslow thickness, mm	
≤ 1	1
1.01–2	59
2.01–4	28
> 4	11
NA^a^	1
Ulceration	
Yes	22
No	77
NA^a^	1
Clark level	
II	3
III	26
IV	57
V	6
Not specified	7
NA^a^	1
Mitotic index, mitoses/mm^2^	
0	8
0.01–0.99	0
1.0–1.99	18
2.0–4.99	41
5.0–10.99	27
11.0–19.99	4
≥ 20	2

^a^One patient had a dermal metastasis considered as the primary site

The 2-day Lymphoseek injection protocol, compared with the 1-day protocol, showed a marginally significant difference (*p* = 0.053) with respect to a reduction of 23% in the total number of SLNs removed per procedure using the GP and PGC.

### Pathology Results

**Fig. 4:**
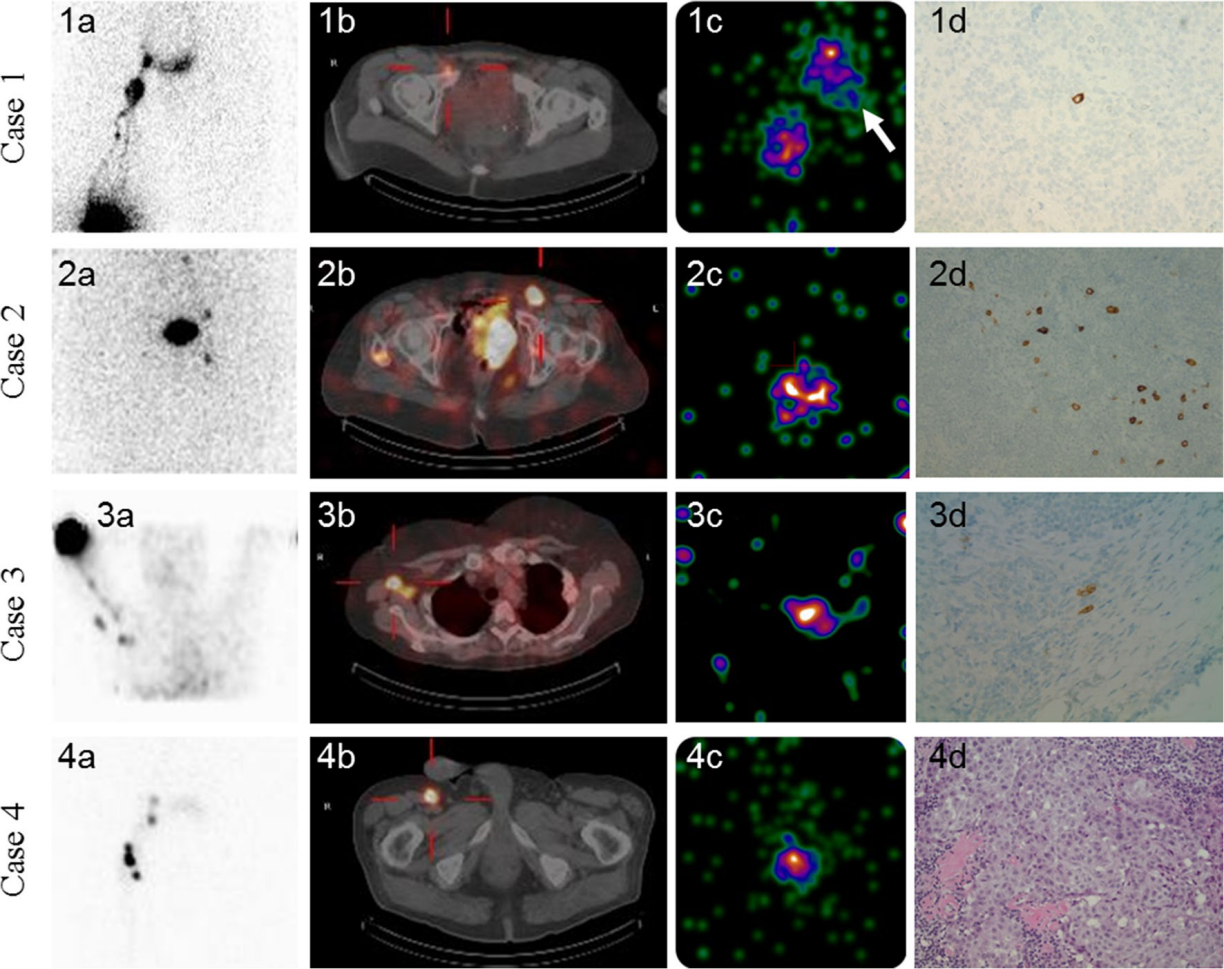
Composite image compilation from the lymphoscintigraphy, SPECT/CT, post-excision PGC image, and SLN micrograph of the four patients in whom the PGC detected additional melanoma-positive SLNs. Case 1: planar lymphoscintigraphy (**1a**) and SPECT/CT (**1b**) visualized two SLNs in the right inguinal and pelvic basins. After conventional surgery, the PGC identified two additional SLNs in the suprainguinal basin and two in the distal external iliac basin. All these SLNs were negative except for one distal external iliac SLN (indicated by an arrow in **1c** and micrograph of **1d**). The removal of this SLN prevented a false-negative procedure. The other SLN in **1c** (not marked by an arrow) was a suprainguinal one, which was negative. Case 2: planar lymphoscintigraphy (**2a**) and SPECT/CT (**2b**) visualized one left femoral  SLN. After conventional surgery, the PGC identified one additional femoral SLN (**2c**), which was removed and upstaged the patient from one to two melanoma-positive lymph nodes (**2d**). Case 3: Planar lymphoscintigraphy (**3a**) and SPECT/CT (**3b**) visualized two SLNs in the right  axilla. After conventional surgery, the PGC identified one additional SLN (**3c**) in the axilla. This SLN was removed, being the only melanoma-positive lymph node (**3d**) preventing a false-negative procedure. Case 4: planar lymphoscintigraphy (**4a**) and SPECT/CT (**4b**) visualized one right femoral SLN. After conventional surgery, the PGC identified one additional femoral SLN (**4c**), which was removed and upstaged the patient from one to two melanoma-positive lymph nodes (**4d**). Thus, PGC upstaged four patients from one to two positive lymph nodes in two patients and from negative to positive SLN status in the remaining two patients. *SPECT* single photon emission computed tomography, *CT* computed tomography, *PGC* portable gamma camera, *SLN* sentinel lymph node

Nodal involvement was identified in 25% (25/100, 95% CI 17–33) of patients, and 11% (35/306, 95% CI 7–14) of the excised SLNs using the GP and palpation. Of the 89 additional SLNs located by PGC, one of the 14 SLNs was tumor-positive where the PGC assisted after ambiguous GP readings. Three of the 75 additional SLNs identified using the PGC after negative GP screening were tumor-positive. Four of the 89 SLNs identified by PGC imaging showed micrometastasis in four patients (Figs. 3 and 4), two of whom had micrometastasis in two SLNs; one tumor-positive SLN was identified by the GP and the other was identified by the PGC. This is clinically significant in removing additional positive SLNs that would otherwise be missed. In another two patients, the only tumor-positive SLN was identified by the PGC, thus preventing two false-negative cases (Fig. 3). Hence, we successfully rejected the null hypothesis of this trial that IDR is zero at a *p* value of 0.000. The overall positive rate of patients was increased when using the PGC, from 25% (25/100) to 27% (27/100). Nodal involvement was identified in 27% (27/100, 95% CI 18–36) of patients, and in 9.9% (39/395) of the excised SLNs. Upper extremity has a lower chance for SLN positivity (*p* = 0.04) compared with the trunk and lower extremity. Other clinical/histological characteristics did not significantly impact SLN positivity. All excised non-SLNs were tumor-negative. In 17 of the 27 patients with positive SLNs, a completion lymph node dissection (CLND) was performed as a secondary procedure.

## Discussion

This unique study design, with the PGC only being used after the GP, allows us to avoid any biases from preoperative PGC imaging, which was performed in previously published studies.^5^^–^7,13 Thus, this approach allows us to make a definitive conclusion regarding the contribution of the PGC in the identification of SLNs. We found that the PGC was able to detect a significant number of additional SLNs (i.e. 89; 23% of the total number of removed SLNs), with four patients having positive SLNs, two of whom were GP-negative and PGC-positive and two were GP-positive and  PGC-positive. After the initial 10–15 cases, as a learning experience, the PGC can be used effectively without significant usage time in the operating room (up to 5 min). In our experience, the device will reduce the operating room surgical time, especially for cases with ambiguous GP readings and complex or uncommon SLN locations.

Since the initial report by Morton et al.,^14^ the FNRs of SSLND worldwide have been reported to be relatively high, ranging between 4.0 and 20%.^15^^–^19 Overall, the reported FNR increased with the length of follow-up.^20^ Various reasons for increased FNR and newer techniques to decrease FNR have recently been further discussed.^21^ This increased FNR in some patients may be due to the fact that nodes with occult micrometastasis were probably missed at the time of SSLND. Failure to detect such positive SLNs may be due to a breakdown in preoperative imaging, intraoperative identification by surgeons, and pathological examination of the SLNs.^21^ Thus, the ‘true’ SLNs may be unidentified. Despite the fact the MSLT-II trial showed no improvement in melanoma-specific survival of CLND for a positive SSLND, melanoma SLN remains an excellent prognostic marker to predict the outcome of patients with primary melanoma.^22^ Furthermore, an unidentified SLN with occult micrometastasis has the potential to negatively impact patient’s prognosis and survival.^18^^,^^23^ Therefore, it is critical to identify melanoma SLNs as accurately as possible to reduce the rate of unidentified positive SLNs. Moreover, Vidal-Sicart et al. were the first to demonstrate that the use of a PGC was helpful in difficult melanoma cases.^6^

Beyond reducing the rate of unidentified SLNs, our study suggested that a PGC is able to identify additional tumor-involved SLNs. Such positive nodes would have ordinarily been resected during the ensuing CLND, following a positive SSLND. However, CLND may be performed less often in the future as the recently published MSLT-II study showed no therapeutic benefit of CLND following randomization of SLN-positive patients to CLND versus observation.^22^ In this study, CLND was performed following the indications prior to the publication of MSLT II.^22^ Nevertheless, improved identification and removal of positive SLNs should still be important goals of SLN surgery.

In addition to its prognostic implication, increased detection of positive SLNs by PGC imaging may have a significant impact in deciding whether adjuvant systemic therapy is indicated, such as nivolumab^24^ and a combination of dabrafenib and trametinib.^25^ Thus, accurate staging by SSLND is crucial in not only rendering accurate staging of melanoma patients but also enabling them to receive effective adjuvant therapy.

The potential weakness of a single-surgeon study design introduces bias into the study; however, the strength of a single-surgeon study design is the standardized intraoperative technique throughout the study. Furthermore, the study does not contain follow-up information of patients in order to provide the FNR in this patient population. Therefore, the follow-up data will be collected prospectively. Because of small sample size, other clinical/histological characteristics did not significantly impact SLN positivity, in contrast to other published studies with a larger patient population showing high-risk features correlating with SLN positivity. Lymphoseek has been shown to detect SLNs with no added benefit of lymphazurin.^10^^,^^26^^,^^27^ Other disadvantages of lymphazurin include its expensive cost and being associated with allergic reactions, although the rate of an allergic reaction is low.^28^ Therefore, in this study, only lymphoseek was used, without lymphazurin.

In summary, to the best of our knowledge we provide the first prospective clinical trial evaluating the added value of a PGC in melanoma surgery. The implications from this study include the following:

- The rate of unidentified positive SLNs has been reduced, and thus may potentially reduce the FNR.
- There has been an upstage from one to two positive lymph nodes in the GP + and PGC + group. In this study, the Breslow is ≥ 1 mm. It has been well-established that the positive patient rate increases with thicker melanomas. Thus, more positive patients with increasing Breslow thickness may be identified.
- From the MLST-II study, less CLNDs would be performed, as mentioned above. Therefore, it is critical to ensure accurate SLN identification in the initial SSLND without retaining positive SLNs to be subsequently removed by CLND.
- Accurate staging of the SLN status is important for consideration of adjuvant therapy of melanoma.

## Conclusions

Intraoperative PGC imaging provides statistically and clinically significant additional information over the conventional method for SSLND. Intraoperative PGC imaging yielded additional SLNs in a significant number of patients compared with GP alone. Furthermore, intraoperative imaging reduces the rate of unidentified positive SLNs in melanoma SLN surgery, which may potentially reduce the FNR.
